# A Rapid and Scalable Method for Multilocus Species Delimitation Using Bayesian Model Comparison and Rooted Triplets

**DOI:** 10.1093/sysbio/syw028

**Published:** 2016-04-07

**Authors:** Tomochika Fujisawa, Amr Aswad, Timothy G. Barraclough

**Affiliations:** ^1^Department of Zoology, Kyoto University, Sakyo, Kyoto 606-8502, Japan;; ^2^Department of Life Sciences, Imperial College London, Silwood Park Campus, Ascot, Berkshire SL5 7PY, UK;

**Keywords:** Bacterial species, Bayesian model comparison, Dynamic programming, Multilocus species delimitation

## Abstract

Multilocus sequence data provide far greater power to resolve species limits than the single locus data typically used for broad surveys of clades. However, current statistical methods based on a multispecies coalescent framework are computationally demanding, because of the number of possible delimitations that must be compared and time-consuming likelihood calculations. New methods are therefore needed to open up the power of multilocus approaches to larger systematic surveys. Here, we present a rapid and scalable method that introduces 2 new innovations. First, the method reduces the complexity of likelihood calculations by decomposing the tree into rooted triplets. The distribution of topologies for a triplet across multiple loci has a uniform trinomial distribution when the 3 individuals belong to the same species, but a skewed distribution if they belong to separate species with a form that is specified by the multispecies coalescent. A Bayesian model comparison framework was developed and the best delimitation found by comparing the product of posterior probabilities of all triplets. The second innovation is a new dynamic programming algorithm for finding the optimum delimitation from all those compatible with a guide tree by successively analyzing subtrees defined by each node. This algorithm removes the need for heuristic searches used by current methods, and guarantees that the best solution is found and potentially could be used in other systematic applications. We assessed the performance of the method with simulated, published, and newly generated data. Analyses of simulated data demonstrate that the combined method has favorable statistical properties and scalability with increasing sample sizes. Analyses of empirical data from both eukaryotes and prokaryotes demonstrate its potential for delimiting species in real cases.

Species constitute the basic taxonomic unit for exchanging information about biological diversity. Defining species boundaries in a consistent manner is therefore of major importance to a broad range of biological disciplines. DNA-based delimitation provides a universal method to detect the signature of species existence applicable to various organisms. Consequently, methods to delimit species from DNA sequences alone have been actively developed over the last decade. For early applications of DNA-based delimitation, available markers were limited to a handful of barcoding loci customized for each type of organism (such as *cox*1 for animals, Hebert et al. 2003), and therefore delimitation methods were designed to handle these single locus sequences (Pons et al. 2006; Puillandre et al. 2012; Fujisawa and Barraclough 2013; Zhang et al. 2013). However, as the cost of sequencing large amounts of DNA has dramatically decreased, and the ease of developing nuclear markers from genome data has increased, the focus has naturally shifted from single to multiple locus approaches.

There has been huge progress recently in the development of statistical methods for multilocus species delimitation, driven by theoretical advances in the multispecies coalescent model (Rannala and Yang 2003; Degnan and Rosenberg 2009). By comparing alternative delimitation hypotheses and finding the best one based on probability distributions of gene trees under the multispecies coalescent model, species can be delimited robustly even with incomplete lineage sorting. Several methods using Bayesian or information theoretic frameworks have been published so far (O'Meara 2010; Yang and Rannala 2010; Ence and Carstens 2011). Empirical studies have evaluated these methods using taxonomically difficult groups (Carstens and Dewey 2010; Hambäck et al. 2013; Satler et al. 2013). Now, the multispecies coalescent model is becoming a standard for multilocus DNA-based delimitation, and there are attempts to integrate these methods with morphology and geography in order to achieve integrative taxonomy (Fujita et al. 2012; Edwards and Knowles 2014).

One drawback of methods based on the multispecies coalescent model is their limited scalability: they rely on the calculation of the probability of obtaining gene trees (or a sequence alignment) given a population tree under the coalescent model, which is relatively time consuming. Also, the joint evaluation of species boundaries and species phylogeny requires searches through an enormous parameter space (Yang and Rannala 2014) , and computation becomes challenging even with small numbers of sampled individuals. Thus, current procedures for multilocus delimitation often require prior assignments of samples to populations, and they are therefore restricted to validation of candidate delimitations based on the assignments. Delimitation without any a priori assignment (species discovery, Ence and Carstens 2011) is feasible only with a limited number of samples, though techniques to reduce search space are being actively studied (Yang and Rannala 2010; Satler et al. 2013). With the increasing ease of sequencing massive multiple nuclear markers (e.g. transcriptome, RAD; Baird et al. 2008; anchored hybrid enrichment; Lemmon et al. 2012), the need for rapid and scalable delimitation methods is becoming more urgent.

An alternative strategy for potentially scalable multilocus species delimitation is to use genealogical concordance. The congruence of between-species branching across gene trees reconstructed for separate loci versus incongruence within species has been used as a signature of reproductive isolation and thereby species diversification (Barraclough et al. 2003). Early attempts that used topological congruence to detect species included the delimitation of cryptic fungi using concordance of gene trees inferred from 5 loci (Koufopanou et al. 1997). The “Genealogical Concordance Phylogenetic Species Recognition” (GCPSR, Taylor et al. 2000) is now commonly used to delimit fungal species which often lack morphological or environmental information (Vialle et al. 2013; Millanes et al. 2014). A disadvantage of using concordance measures between multiple gene trees is that it is hard to treat them under statistical models of evolution. It has been known that a set of multiple gene trees do not necessarily “concord” with each other even if they are generated under the same species tree because of the stochastic nature of the coalescent process. Moreover, the consensus topology of gene trees may not be congruent even with the species tree that generated the gene trees (the anomalous gene tree problem, Degnan and Rosenberg 2006). Thus, the degree of concordance at which one can confidently infer species is not as simple as first perceived. Modeling the distribution of congruence of trees is intrinsically difficult as it must incorporate calculations of the probability of gene trees under a given species tree. Only 1 nonparametric method with a simulated null model has been devised for statistical delimitation based on topological congruence (O'Meara 2010).

Here, we develop a new method for multilocus species delimitation using gene tree congruence, which employs a likelihood model based on the distribution of triplets. We define a triplet as a partial rooted tree consisting of 3 tips. Using the distribution of rooted triplets is a promising approach to model congruence between gene trees under the coalescent framework for 2 reasons. First, the number of triplets with congruent topology is an intuitive measure of topological similarity between trees. Second, the distribution of triplets is readily tractable under the multispecies coalescent framework (Pamilo and Nei 1988) and has been used successfully for rapid inference of phylogenetic trees (Liu et al. 2010). The distribution model for triplet topology is simple and can be extended for intuitive and rapid model-based delimitation. We tested the performance of the new method with various data sets including simulated gene genealogies and both published and newly generated sequence data from both eukaryotes and microbes. The method provides a tractable approach for multilocus delimitation that is scalable to samples with hundreds of individuals across large clades.

## Methods

### Calculation of the Likelihood of Triplet Distributions

We employ common assumptions of the multispecies coalescent model (Rannala and Yang 2003; Degnan and Rosenberg 2009): there is neutral random coalescence without structure within species (i.e., panmixia), no gene flow or horizontal transfer between species, and loci evolve independently without intra-locus recombination. In addition, to simplify, we assume initially that the topology of the gene tree is known without error. Under these assumptions, the distribution of triplet topologies is modeled by a simple trinomial distribution as follows.

A bifurcating tree with K tips can be decomposed into $\left(\begin{array}{c} K\\ 3 \end{array}\right)$ rooted triplets. For a given triplet of 3 individuals, a, b, and c, there are 3 possible topologies, ab|c, ac|b, and bc|a. When genealogies from N independent loci are sampled, the numbers of gene trees that conform to each topology—represented by $n_{\text{1}}$, $n_{\text{2}}$, and $n_{\text{3}}$—are modeled by a trinomial distribution for each triplet. When individuals a, b, and c belong to a single species, then under our assumption that the species is panmictic, there is an equal probability of observing each of the 3 triplet topologies because coalescent events of any pair are equally likely in a panmictic population. Therefore, the distribution of counts of the 3 topologies is represented by an equiprobable trinomial distribution with likelihood:

(1) $$P_{W}\left(n_{1},n_{2}n_{3}\right)=\frac{N}{n_{1}!n_{2}!n_{3}!}{\left(\frac{1}{3}\right)}^{N}.$$

When individuals are sampled from 2 or 3 distinct species, under the assumptions of the multispecies coalescent process above, the probability of observing triplets congruent with the species tree topology is ${\text{1-2e}}^{\text{-}𝛌}$/3, where 𝛌 is the length of the internal branch measured by coalescent time units on the species tree, and the probability of observing an incongruent triplet is ${\text{e}}^{\text{-}𝛌}$/3 (Pamilo and Nei 1988; Degnan and Rosenberg 2006). Hence, the distribution of triplet counts follows the skewed trinomial distribution.

(2) $$\begin{array}{l} P_{B}\left(n_{1},n_{2},n_{3}|v=n_{1},𝛌\right)\\ \;=\frac{N}{n_{1}!n_{2}!n_{3}!}{\left(1-\frac{2}{3}e^{-𝛌}\right)}^{n_{1}}{\left(\frac{1}{3}e^{-𝛌}\right)}^{n_{2}+n_{3}}. \end{array}$$

In the equation above, $𝛎=n_{\text{1}}$ is the count of triplets congruent with the species tree topology (dominant topology), whereas $n_{\text{2}}$ and $n_{\text{3}}$ denote the counts of incongruent triplets (minority topologies). Note that this distribution does not distinguish the 2-species case from the 3-species case. Therefore, it is impossible to split a pair of species only represented by 2 samples but possible to split a species represented by a single sample from species with 2 or more samples.

### A Bayesian Model Comparison Framework

In the absence of prior knowledge of the species tree, the observer cannot know a priori which triplet is the triplet concordant with the species tree. Choosing the most frequently observed triplet and using its count as 𝛎 in the above equation introduces a bias toward the 3-species case and increases the rate of false positives (Supplementary Figure S1 in Supplementary Material, available at Dryad at http://dx.doi.org/dryad.3cb25). We therefore develop a Bayesian model comparison framework to take the unknown species tree into account.

When the species tree is unknown, there are 3 models that conform to the 3-species case described above. We call these 3 models, ${\text{T}}_{B}=\{\tau_{b1},\tau_{b2},\tau_{b3}\}$, each of which is associated with 1 of 3 possible topologies of the underlying species tree. The likelihood functions of the models in ${\text{T}}_{B}$ are described by $P_{B}$ in Equation (2), with the dominant triplet 𝛎 matching $n_{\text{1}}$, $n_{\text{2}}$, and $n_{\text{3}}$ for $\tau_{\text{b1}}$, $\tau_{\text{b2}}$, and $\tau_{\text{b3}}$, respectively.

We also consider 3 models for the case of a single species, following the scheme of Yang and Rannala (2014). We call the set of the 3 models, ${\text{T}}_{W}=\{\tau_{w1},\tau_{w2},\tau_{w3}\}$. Each model in ${\text{T}}_{W}$ is again associated with 1 of the 3 possible topologies and has its counterpart in ${\text{T}}_{B}$ (Yang and Rannala 2014). The likelihood functions are ${\text{P}}_{W}$ in Equation (1) and they are identical across models.

With the 6 candidate models, the joint posterior probability of τ (model) and 𝛌 (branch length) given triplet counts $X=(n_{\text{1}},n_{\text{2}},n_{\text{3}})$ is

(3) $$P(𝛌,\tau |X)=\frac{P(X|𝛌,\tau )\pi (𝛌)\pi (\tau )}{\int \sum_{\tau \in {\text{T}}_{B}\cup {\text{T}}_{W}}P(X|𝛌,\tau )\pi (𝛌)\pi (\tau )d𝛌},$$

where π(τ) and π(𝛌) are prior probabilities of τ and 𝛌. We obtain the posterior probability of τ by marginalizing the joint posterior by 𝛌.

(4) $$P\left(\tau |X\right)=\frac{\int P(X|𝛌,\tau )\pi (𝛌)\pi (\tau )d𝛌}{\int \sum_{\tau \in {\text{T}}_{B}\cup {\text{T}}_{W}}P(X|𝛌,\tau )\pi (𝛌)\pi (\tau )d𝛌}.$$

To simplify the expression for the posterior, we now employ simple uniform priors, $\pi \left(\tau \right)=\frac{1}{6}$ and π(𝛌)=1L[0≤𝛌≤L]. We use a prior range up to L=5 throughout this study, which covers a realistic range of frequency of dominant triplets, 0.33 ≤ ${\text{1-2e}}^{\text{-}𝛌}$/3 ≤ 0.996. The posterior probability of the model with the uniform priors is,

(5) $$P\left(\tau |X\right)=\frac{\int_{0}^{L}P(X|𝛌,\tau )d𝛌}{\int_{0}^{L}\sum_{\tau \in {\text{T}}_{B}\cup {\text{T}}_{W}}P(X|𝛌,\tau )d𝛌}.$$

The integration of P(X|𝛌,τ) over 𝛌 has a tractable analytical solution, therefore a reversible jump Markov chain Monte Carlo (MCMC) is not required to characterize this posterior distribution. When τ is 1 of the 3 models of ${\text{T}}_{W}$, the integration over 𝛌 is trivial.

$$\int_{0}^{L}P\left(X|𝛌,\tau \in {\text{T}}_{W}\right)d𝛌=L\cdot P_{W}(X).$$

When τ belongs to ${\text{T}}_{B}$, the integration of the likelihood function is represented by the incomplete beta function. When the dominant triplet 𝛎 is $n_{\text{1}}$,

$$\begin{array}{l} \int_{0}^{L}P\left(X|𝛌,\tau \in T_{B}\right)d𝛌\\ \;={\left(\frac{1}{2}\right)}^{n_{2}+n_{3}}C\int_{\frac{2e^{-L}}{3}}^{\frac{2}{3}}{\left(1-x\right)}^{n_{1}}x^{n_{2}+n_{3}-1}dx={\left(\frac{1}{2}\right)}^{n_{2}+n_{3}}\\ \;C\left\{𝛃\left(\frac{2}{3};n_{2}+n_{3},n_{1}+1\right)-𝛃\left(\frac{2e^{-L}}{3};n_{2}+n_{3},n_{1}+1\right)\right\}, \end{array}$$

where 𝛃(x;a,b) is the incomplete beta function and C is the multinomial coefficient in Equation (2). Replacing $n_{\text{1}}$ with $n_{\text{2}}$ or $n_{\text{3}}$ gives solutions for $𝛎=n_{\text{2}}$ or $n_{\text{3}}$.

The models in ${\text{T}}_{B}$ are supporting the 3-species delimitation, B (i.e., samples are from 3 distinct species); therefore the posterior probability of the delimitation B is a sum of the 3 posterior probabilities of the models in ${\text{T}}_{B}$.

(6) $$P\left(B|X\right)=\frac{\sum_{\tau \in {\text{T}}_{B}}\int_{0}^{L}P\left(X|𝛌,\tau \right)d𝛌}{\sum_{\tau \in {\text{T}}_{B}}\int_{0}^{L}P\left(X|𝛌,\tau \right)d𝛌+3L\cdot P_{W}(X)},$$

and the posterior probability of the single-species case delimitation, W (samples are from a single species) is,

(7) $$P\left(W|X\right)=\frac{3L\cdot P_{W}(X)}{\sum_{\tau \in {\text{T}}_{B}}\int_{0}^{L}P\left(X|𝛌,\tau \right)d𝛌+3L\cdot P_{W}(X)}.$$

With a given hypothesis of delimitation, each of the $\left(\begin{array}{c} K\\ 3 \end{array}\right)$ triplets is assigned to 1 of the 2 categories defined above, that is, a, b, and c either belong to the same species or to multiple species. The overall posterior probability of a given delimitation for all K taxa is the product of the posterior probabilities of all triplet counts of 2 categories. For a set of triplet counts, ***w***, which is assigned to delimitation W, and a set ***b***, which is assigned to delimitation B, the log-posterior probability of a delimitation D is as follows.

(8) $$\begin{array}{l} logP\left(D|X\right)=\sum_{\left(n_{1},n_{2},n_{3}\right)\in w}logP(W|n_{1},n_{2},n_{3})\\ \;+\sum_{(n_{1},n_{2},n_{3})\in b}logP(B|n_{1},n_{2},n_{3}). \end{array}$$

We use this quantity as the posterior probability score. Note that it is not a true posterior probability of delimitation because it ignores the mutual dependence of the parameters of the triplet distribution caused by overlapping membership of some triplets. However, the similar approximation of likelihood functions has been used successfully in statistical phylogenetic inference (Liu et al. 2010) and we test its performance by simulation here.

### Finding the Best Delimitation Model

The posterior probability score described above is used to find the optimal delimitation from a set of delimitations of samples. The number of all possible delimitations of K samples is represented by the Bell number, $\sum_{i=1}^{K}\left\{\begin{array}{c} K\\ i \end{array}\right\}$ (Bell 1934), where $\left\{\begin{array}{c} K\\ i \end{array}\right\}$ is a Stirling number of second kind, defined as the number of all possible ways to split K items into i groups. This number is common to partitioning problems and intractably large. An approach taken to reduce the number of delimitations considered is using a guide tree (Yang and Rannala 2010), which gives a hierarchical structure of the multiple delimitations. Different combinations of splitting and lumping of lineages on a given guide tree are searched to find the best delimitation. Conventional search methods with the guide tree approach use either reversible-jump MCMC for characterizing posterior probabilities of competing delimitations (Yang and Rannala 2010, 2014) or heuristic search algorithms to find the optimal combinations of splits and lump of lineages (O'Meara 2010; Satler et al. 2013).

We now consider only the problem of finding the best delimitation on a fixed guide tree without tree rearrangement. The number of all possible delimitations under a given guide tree with S tips is approximately $\left\lfloor 1.5^{S}\right\rfloor$ (floor of $\text{1}.{\text{5}}^{S})$ in the worst case and S in the best case (Fujisawa and Barraclough 2013). The size of the space is reduced compared with the Bell number, but still grows exponentially with the number of species in the worst case. We developed a new dynamic programming algorithm to rapidly find the best combination of lineages on the guide tree, taking advantage of the optimal substructure of the likelihood model and the guide tree structure.

**Figure 1.**
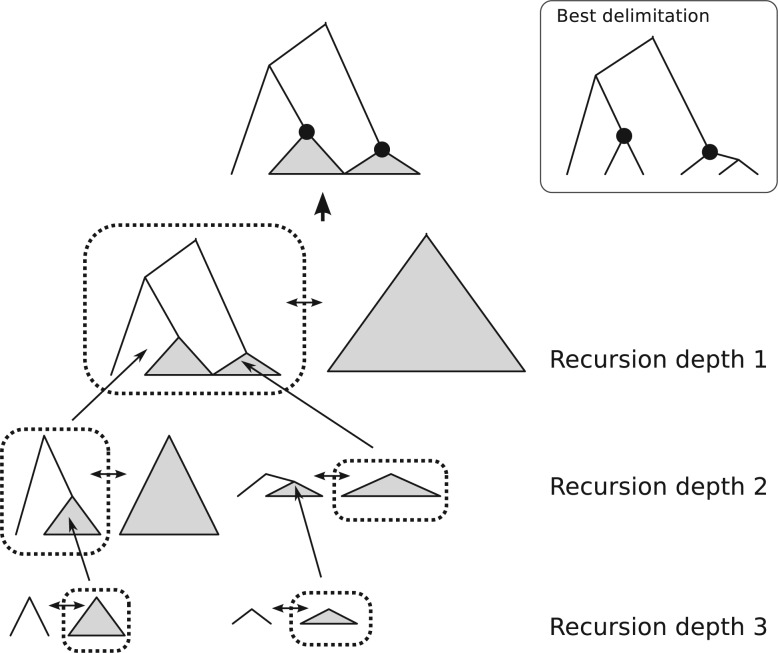
An illustration of how the dynamic programming algorithm finds the optimal delimitation. Below each node in the guide tree, 2 alternative delimitations are compared (horizontal arrows) and the better one is chosen (dotted squares). The best delimitation below 1 node is inserted into the comparison at the higher level successively to yield the final optimal delimitation.

Given a delimitation under a guide tree, D, its posterior probability score can be decomposed into a sum of the scores of the delimitations of 2 subtrees descending from the root, ${\text{logP(D}}_{\text{L}})$ and $\text{logP}({\text{D}}_{R})$, and a constant factor because Equation (8) is additive.

$$logP\left(D\right)=logP\left(D_{L}\right)+logP\left(D_{R}\right)+c$$

(9) $$c=\sum_{(n_{1},n_{2},n_{3})\in b_{root}}logP(B|n_{1},n_{2},n_{3}),$$

where c is a constant representing a score for triplets crossing over 2 subtrees descending from the root node of the guide tree. Because triplets are not shared between subtrees and their posterior probabilities are independently calculated, the optimal solutions for each subtree must be included in the global solution. Therefore, finding the global optimal solution can be reduced to finding solutions to subtrees' delimitations, and iteratively solving and combining them yields the global solution. An exception is the case where logP(D) is represented by the root of the guide tree; that is, all samples are from the same species. In the case of root delimitation, Equation (9) does not hold because the constant of the third term must be represented by P(W|X) not P(B|X). So, the dynamic programming algorithm must compare the “root” delimitation with the aggregated solution of subtrees in each step. This leads to the algorithm described in Figure 1 and Supplementary Text S1 in Supplementary Material. This algorithm calculates the global optimal posterior probability score from a guide tree, and the best delimitation was obtained by keeping the set of nodes producing the best score. The algorithm reduces the number of likelihood calculations to twice the number of the nodes on the guide tree.

We implemented the method in a program called “tr2” (Trinomial distribution of Triplets) for calculation of posterior probability scores for delimitation hypotheses and the search algorithm for the best delimitation given a guide tree. The program is implemented in Python and can run on any operating system (Distributed at https://bitbucket.org/tfujisawa/tr2-delimitation).

### Simulations and Case Studies

We used simulated and real gene trees to test the performance of the method. First, we performed coalescent simulations with species trees with 3 and 10 tips. Then, we analyzed a published data set of rattlesnakes with 29 individuals and a newly sequenced data set of 144 *Bacillus cereus* isolates.

#### Three-species simulations

**Figure 2.**
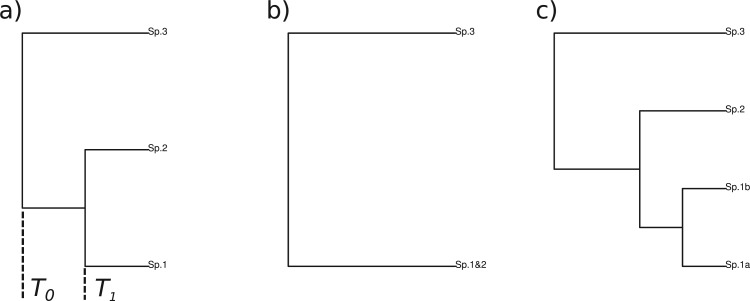
Schematic representations of alternative hypotheses of delimitation for the 3-species simulations: a) correct hypothesis, b) under-split, and c) over-split.

In order to test the performance of the delimitation model, we first conducted a simple 3-species simulation and assessed the error rates of the model. In this simulation, we assume gene trees are known without error. Gene genealogies were simulated within a species tree with 3 tips and fixed branch lengths, ${\text{T}}_{1}=4000$ and ${\text{T}}_{0}=8000$ generations (Fig. 2a). The number of samples per species was set to 10, totalling 30 individual samples. The effective population sizes were set to 1/2*${\text{T}}_{1}$ to 8*${\text{T}}_{1}$ for all species (${\text{T}}_{1}=1/8$Ne – 2Ne generations). Coalescent trees within the species tree were simulated using SIMCOAL (Excoffier et al. 2000) assuming that 1 species represents 1 population and populations merge on speciation events. Custom scripts were used to generate input files for SIMCOAL from species trees. Twenty-five independent loci were simulated 100 times, which resulted in 2500 gene trees in total. The posterior probability for a global delimitation W (all individuals are from a single species) and the 3 alternative models representing correct delimitation (a), under-splitting (b) and over-splitting (c) (Fig. 2) were calculated with the increasing numbers of loci between 5 and 25 with step 5. Error rates, that is, the frequency of choosing an incorrect model as the best model, were recorded for each iteration.

#### Ten-species simulations

The ten-species simulation considers more realistic conditions. Species trees with 10 tips were simulated under the Yule model with a constant speciation rate. The total depth of species trees, T, was rescaled to 20,000 generations, and the effective population size (Ne) of species was set as 1, 1/2, 1/4, 1/8, and 1/16 times of T (Ne = 1250–20,000). These parameter settings cover speciation rates and effective population sizes observed in various eukaryotic groups (Coyne and Orr 2004; Charlesworth 2009) including extreme cases of rapid radiations. Gene trees with 10 samples per species (100 total samples) for 40 independent loci were simulated using SIMCOAL and the custom scripts. Simulations were replicated 100 times.

In the first simulation, hereafter simulation A, we assume that the topology of the guide tree and assignment of terminals to species groups is known. This simulation tests whether the method can correctly find the positions of nodes which define species from multiple competing combinations on a guide tree. The tr2 program was run with the species tree as a guide and simulated gene trees as inputs. The number of loci used ranged from 5 to 40.

In the second simulation, B, delimitation was conducted solely from sets of gene trees (species discovery approach). A consensus tree was built from gene trees from multiple loci using the rooted triple consensus (Ewing et al. 2008). Then, the consensus tree was used as the guide tree in the delimitation step. This guide tree contains all possible hierarchical delimitations, from each individual representing a separate species to all individuals representing a single species. Polytomies on consensus trees were randomly resolved by the “multi2di” function in the “ape” package (Paradis et al. 2004). In addition, we performed a set of simulations to assess the effect of increasing numbers of loci and individual samples. Gene trees were simulated within the same species trees as above with Ne = T/4, but the total number of samples was reduced to 50 (5 per species) and the number of loci was doubled, keeping the total sample size (number of loci × number of samples) constant. Delimitation with tr2 was conducted in the same procedure as simulation B.

The third simulation, C, considers conditions where gene trees and species trees are estimated from DNA sequences. Sequences were simulated along the branches of the gene trees simulated above using Seq-Gen (Rambaut and Grassly 1997) assuming HKY+G model (Ts/Tv = 2.5 and α=0.1) and 3% of overall genetic variations. These parameters were chosen to be comparable to the case studies described in the next sections. Sequence length was set to a constant length of 750 bp. Gene trees were reconstructed from the simulated sequences using RAxML with a GTR+G model (Stamatakis 2014) and rooted by the “-I f” option of RAxML. Guide trees were estimated from the reconstructed gene trees with the rooted triple consensus, and delimitation was conducted with tr2. Under the parameter settings above, within-species genetic variation of simulated sequences ranged from 0.3% to 1.4% depending on Ne, and between-species variation was 3.0%.

The number of estimated species and the number of exact matches between estimated and true species were measured as the accuracy of delimitation. The elapsed time for each trial was also recorded. The numbers of nonmonophyletic species were counted to measure the degree of incomplete lineage sorting. The effects of Ne, the number of loci, simulation type (A, B, and C) and their second interaction terms on the proportion of exact matches were tested using GLM. For simulation B, the effect of the 2 sampling strategies was also tested. Simulations and delimitations were run on a Linux personal computer with a 2.3 GHz Intel i5 quad-core processor and 4 GB memory.

#### Case study 1: Sistrurus Rattlesnakes

Kubatko et al. (2011) sampled 18 nuclear loci and 1 mtDNA locus of *Sistrurus* rattlesnakes. The data set of the nuclear loci included 58 phased sequences from 29 individuals of 6 known subspecies of *S. catenatus*/*S. miliarius* and 2 outgroups. Kubatko et al. (2011) reported that 1 subspecies, *S. catenatus catenatus*, exhibited signatures of a distinct species status, whereas the other 5 subspecies did not show significant evidence of independent species based on the monophyly-based test described by Rosenberg (2007). We reanalyzed this data set. The gene trees and an alignment matrix of 18 nuclear loci were downloaded from TreeBase (accession: TB2:S11174). The trees were randomly resolved with “multi2di.” Then, a consensus tree was built using the rooted triple consensus from them, and the best delimitation was determined with the consensus as the guide tree. A resampling procedure of loci was conducted by progressively adding single loci in random order. Polytomies were randomly resolved in each iteration. The resampling was repeated 50 times to characterize the effect of increasing number of loci on the delimitation. Genetic variation within subspecies was 0.2% and between species 2.2%.

#### Case study 2: Bacillus Multilocus Sequence Typing

We tested the applicability of the tr2 method to bacterial species using a multilocus sequence typing (MLST) data set of the *B. cereus* complex. MLST is a typing scheme for bacterial species/subspecies using a few (typically 7) loci (Maiden et al. 1998; Maiden 2006). It is widely used in clinically relevant bacteria and occasionally in environmental prokaryotes to delimit species (e.g., Papke et al. 2007). Although bacterial reproduction is largely clonal, in many bacteria including *B. cereus*, genetic exchange also occurs (Vos and Didelot 2009). If there was frequent gene exchange within a group of closely related individuals, but none between distantly related groups, this could lead to units equivalent to reproductively isolated species in sexual eukaryotes (Didelot et al. 2011; Barraclough et al. 2012).The tr2 method should be able to delimit such a group as a putative species. However, in clonal bacteria without any recombination, the delimitation method based on gene tree congruence would delineate all individuals as separate species because the true genealogy of each locus would be identical. Another complication is that horizontal transfer might occur rarely between otherwise distinct species. This could introduce additional incongruence among loci between otherwise separate species. We were interested to see how the method coped with a prokaryotic clade that might display these complications.

Our sample comprised 144 isolates originally collected from evenly spaced quadrats in the walled garden at Silwood Park for the study by Collier et al. (2005). In brief, freezer isolates were regrown on *B. cereus* selective agar and DNA extracted using Chelex Instagene matrix method. The 7 house-keeping genes used for standard *B. cereus* MLST (Jolley et al. 2004) were PCR amplified and Sanger sequenced using primers and conditions at the MLST database (http://pubmlst.org/bcereus/info/primers.shtml). Sequences were edited in Geneious and trimmed to the lengths used at the MLST database. Full details are provided elsewhere (Collier et al. 2005; Barraclough et al. in preparation), and sequences are available at GenBank (Accession: KT806485-KT807462).

Alignment lengths of the MLST sequences ranged from 348 bp to 504 bp, and there were 29–55 unique haplotypes at each locus (maximum of 55 for purH and minimum of 29 for glpF and gmk). The complete data matrix excluding missing loci contained 2806 bp from each of 114 isolates, which included 99 unique multilocus sequence types. Overall genetic variation was 4.0%. Sequences from the 7 loci were separately aligned with MUSCLE 3.8 (Edgar 2004). Gene genealogies of the 7 loci were estimated using BEAST 1.80 (Drummond et al. 2012). Ten million generations of MCMC sampling were run with a GTR+G substitution model and the log-normal relax clock model (Drummond et al. 2006). Twenty percent of the MCMC samples were discarded as burn-in. The convergence of the parameters was checked by effective sampling size using Tracer (Rambaut and Drummond 2007), and the maximum clade credibility trees (MCC trees) were extracted from the MCMC runs using TreeAnnotator.

Two methods were used to obtain guide trees for delimitation of the *Bacillus* group. First, a consensus tree was constructed using the rooted triple consensus from the MCC trees of 7 loci. Second, in order to account for the effects of horizontal transfer on the guide tree estimation, we ran ClonalFrame (Didelot and Falush 2007) on the concatenated alignment. ClonalFrame estimates the most likely clonal genealogy by removing putative horizontally transferred regions. An MCMC of ClonalFrame was run with 800 thousand generations, and 50% of the chain was discarded as burn-in. Convergence of parameters was examined by checking effective sample size using Tracer. The 50% majority consensus from the ClonalFrame MCMC was used as a second guide tree. Resampling of loci was conducted 50 times using these 2 guide trees. To further account for the uncertainty of tree building, 100 trees were sampled from the MCMC chain from BEAST for each locus and from the chain of ClonalFrame, and delimitation was repeated with these 100 sets. The frequency for each pair of samples to be grouped in the same species was recorded. (Sequence alignments and trees are available at Dryad: http://dx.doi.org/10.5061/dryad.3cb25).

## Results

### Three-Species Simulations

**Figure 3.**
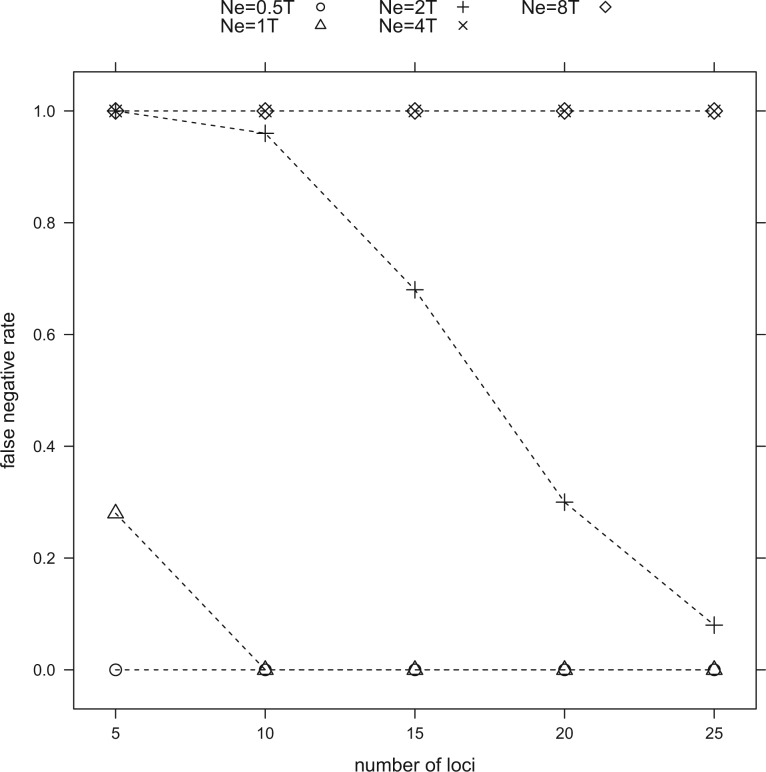
Relationships between false negative rate and the number of loci used for delimitation in the 3-species simulations that simulated different effective population sizes within species relative to the divergence time between species.

The overall false positive rate (FPR, rate of over-splitting) in the 3-species simulations is 0.0 in all iterations with all numbers of loci between 5 and 25. False negative rates (FNR, rate of under-splitting) decrease as the number of loci used increases (Fig. 3). FNR of less than 30% were attained with only 5 loci when Ne was 2000 and 4000 (equivalent to T = 2Ne and Ne), whereas the FNRs reached 30% with 20 loci when Ne was 8000 (T = 1/2Ne). With larger Ne values, the decrease of FNRs was much slower, and the method was not able to correctly delimit species within the range of loci used in the simulations when Ne was 16,000 and 32,000 (T = 1/4Ne-1/8Ne). The average time required for 1 trial was 0.5 s.

### Ten-Species Simulations

**Figure 4.**
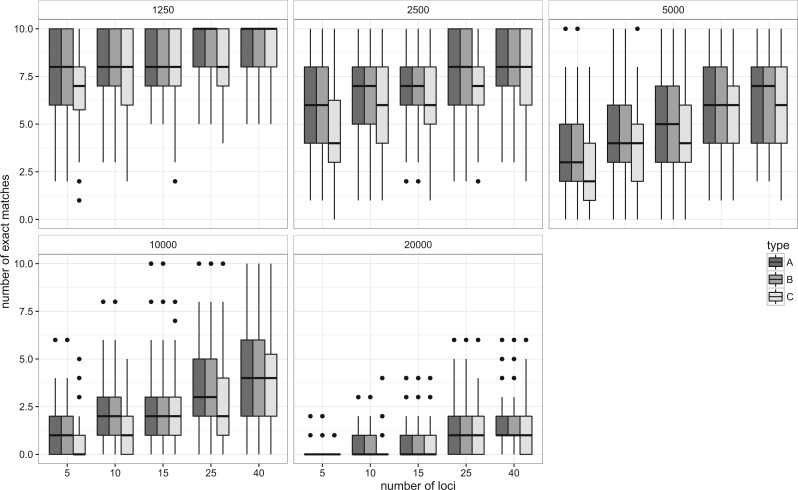
Relationships between the number of exact matches and the number of loci used in the 10-species simulations. A) Both guide trees and gene trees are known, B) guide trees are estimated but gene trees are known and C) guide trees and gene trees are estimated from DNA sequences.

**Figure 5.**
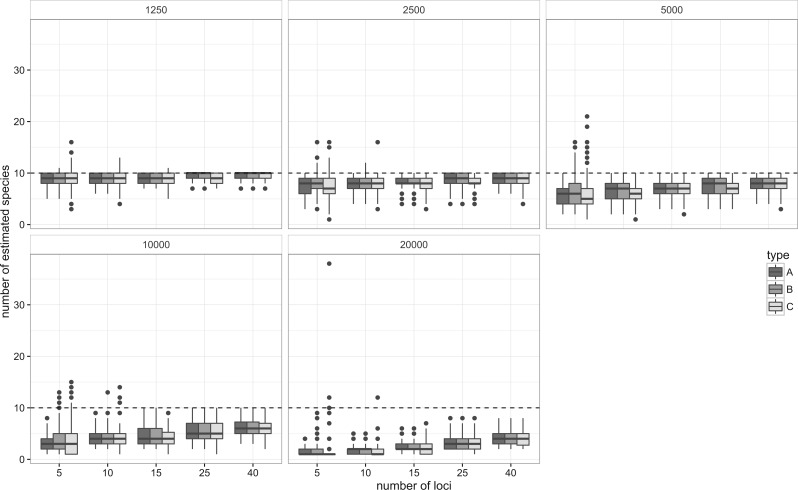
Relationships between the number of estimated species and the number of loci used in the 10-species simulations. A) Both guide trees and gene trees are known, B) guide trees are estimated but gene trees are known and C) guide trees and gene trees are estimated from DNA sequences.

When true species trees are given as the guide tree, the method appeared to delimit species consistently. The proportion of exact matches increased with the number of loci used (Fig. 4, A), and the number of estimated species approached the true number of species, 10 (Fig. 5, A). With low Ne value (Ne = 1250), the median number of exact matches reached 10 when 25 or more loci were used. The increase in the number of exact matches slowed down with larger Ne values, for example, when Ne ⩾ 5000, 40 loci were not enough to attain 100% exact matches.

The accuracy was slightly reduced when the guide trees were estimated by the consensus method (Figs. 4 and 5, B). However, the effect of simulation type was not significant (z=−0.33, P=0.74 for simulation type, GLM with binomial errors), whereas Ne and the number of loci were highly significant (P<​​<0.001 for both Ne and the number of loci). In addition to the under-split observed in the simulation A, a few over-splits occurred especially when the number of loci was small. In 0.9% of trials, the method estimated more than 10 species. Overall accuracy still increased when more loci were added. When the gene trees were estimated from the simulated sequences, the accuracy further decreased, especially when the number of loci was small (Fig. 4, C). The accuracy was significantly lower than other simulation types (z=−4.42, P<​​<0.001, GLM with binomial error). Even more frequent over-splits were observed: the number of trials with >10 estimated species reached 2.0% (Fig. 5, C).

The time required for a delimitation process increased nearly linearly with the number of loci (Supplementary Figure S2 in Supplementary Material). Median time ranged from 23 s to 47 s for 10 tip guide trees and from 135 s to 162 s for guide trees with 100 tips. Average proportions of nonmonophyletic species were between 0.34 for Ne = 1250 and 0.97 for Ne = 20,000 (Supplementary Figure S3 in Supplementary Material), indicating nonmonophyly is prevalent even for small Ne values. The accuracy of delimitation was significantly lower when fewer loci and more samples were used (z=−3.27, P=0.001, GLM with binomial error, Supplementary Figure S7 in Supplementary Material).

### Rattlesnakes

**Figure 6.**
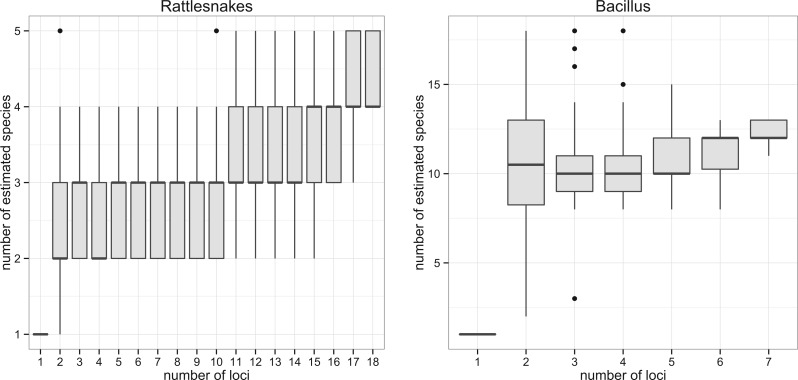
The number of species estimated when randomly resampling loci in the empirical data sets. a) Rattlesnakes. b) *Bacillus* complex.

The method delimited 4 putative species of the *Sistrurus* rattlesnakes, including 2 in-group and 2 out-group species (Fig. 6a, Supplementary Figure S4 in Supplementary Material). The 2 in-group species matched with the known taxonomic species, *S. catenatus* and *S. miliarius*. Random resampling of loci indicated the number of estimated species does not saturate within the range of loci used in this study (Fig. 6a). With 18 loci, 28% of repeated delimitations split *S. catenatus* into 2 groups: 1 group exclusively consisted of a subspecies *S.c.catenatus* and another group consisted of *S.c.edwardsii* and *S.c.tergeminus*. Three subspecies of *S. miliarius* were always grouped together into a single species.

### Bacillus MLST

**Figure 7.**
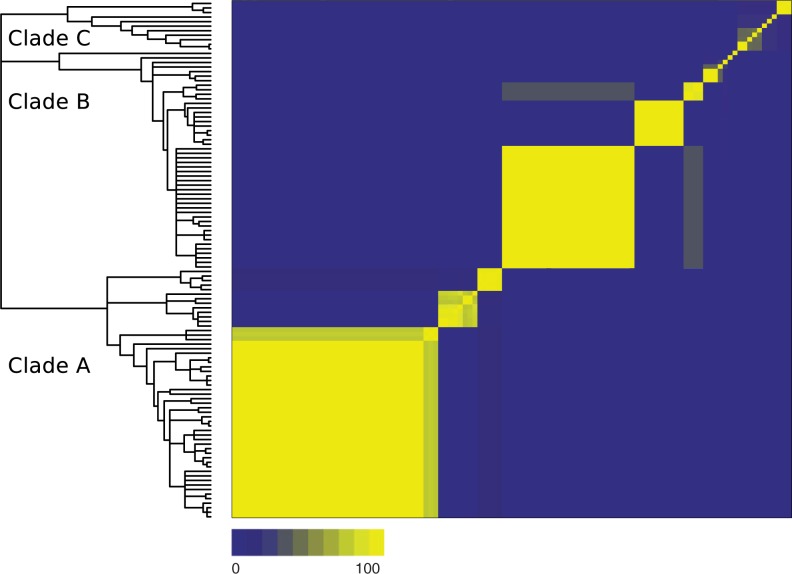
Results of delimitation with 100 sets of gene trees and guide trees sampled from MCMC runs. Trees from ClonalFrame MCMC were used as guide trees. a) The 50% majority consensus tree built with ClonalFrame. b) The frequency that each pair of isolates was grouped by tr2.

Delimitation using the 7 MCC trees and rooted triple consensus tree resulted in 7 putative species, whereas the delimitation with ClonalFrame consensus resulted in 11 species. The majority of nodes on the rooted triple consensus were unresolved (Supplementary Figure S5 in Supplementary Material). ClonalFrame robustly recovered 3 clades, 2 of which were unresolved in the rooted triple consensus (Clade A, B, and C in Fig. 7). The difference between the 2 approaches is consistent with horizontal transfer affecting topologies deeper in the tree; we mainly focus on the result of delimitation using the ClonalFrame guide tree. Resampling of loci showed that there was substantial variation in the number of estimated species (Fig. 6b): the sample of 7 loci might be too few for robust delimitation in this case. Repeated delimitations run on 100 sets of MCMC tree samples exhibited 18 species that were consistently delimited (Fig. 7). Although clades A and B were grouped into 3 or 4 large clusters, clade C was more frequently separated into small singleton species. Frequencies for isolates to be grouped in species with other isolates within these clades were on average 61% and 40% for clade A and B and 35% for clade C.

We estimated linkage disequilibrium (LD) within subsets of these groups to test for variation in recombination rate. Samples were taken from within the largest clusters in clade A and B respectively, and randomly from within clade C, and LD of variable sites was calculated for each group by the “LD” function of an R package “pegas” (Paradis 2010). The test calculates the correlation between pairs of variable sites (Zaykin et al. 2008). There are distinctive linkage patterns between and within the 7 loci in the 3 groups (Supplementary Figure S6 in Supplementary Material). In clade A and B, strong to moderate LD within each locus and LD between a few pairs of loci were observed, but LDs between loci were small (Median within-locus $R^{\text{2}}=0.49$ and 0.16 and Median between-locus $R^{\text{2}}=0.07$ and 0.08 for clades A and B, respectively). This is consistent with recombination among separate loci, but linkage within loci. On the other hand, in clade C, there were moderate or high levels of LD between most loci (Median within-locus $R^{\text{2}}=0.38$ and between-locus $R^{\text{2}}=0.29$ for clade C), consistent with low rates of recombination even between loci.

## Discussion

Congruence between gene trees provides intuitive and readily tractable statistical models for multilocus species delimitation. In this article, we developed a method to delimit species based on topological congruence or incongruence of triplets quantified by 2 types of trinomial distribution models. These models were derived from the multispecies coalescent framework and can be used for robust delimitation of species from gene trees with incomplete lineage sorting. The simulation studies confirmed that the method can consistently delimit species without monophyly, and its performance increased with the number of loci and decreased with larger effective population size relative to divergence time.

The accuracy of the method is slightly lower than the reported performance of conventional multilocus delimitation methods (Camargo et al. 2012); more than 25 loci were required to delimit with 95% success rate under the condition T = 0.5*Ne (Fig. 2), whereas Camargo et al. (2012) reported 60–100% success with 10 loci by conventional methods. The advantage of tr2 appears to be its speed and applicability to large data. According to Camargo et al. (2012), with a 4 species guide tree, SpedeSTEM (Ence and Carstens 2011) ran in 30 s with 20 samples and BP&P (Yang and Rannala 2010) with 80 samples in 6.5 h. The order of speed of the tr2 (30 s with 100 samples and 10 species with known gene trees) is comparable to the fastest conventional method. When a sequence alignment is used, additional time for tree reconstruction is required (e.g. ∼1 min per locus by RAxML), but the reconstruction–delimitation procedure can still scale to large data sets. In addition, the dynamic programming algorithm finds the global solution on a given guide tree, whereas most heuristic optimizations do not guarantee it. The method was sufficiently conservative to over-splitting, which is a favorable property for DNA-based species delimitation methods (Carstens et al. 2013).

The simulation studies also showed that the accuracy of the guide tree is crucial for accurate multilocus delimitation. It has been known that the incorrect assignment of samples on guide trees results in over-splits of species in multilocus delimitation (Leaché and Fujita 2010; Zhang et al. 2014). The over-splits observed in the discovery approach (simulation B and C) are likely to have resulted from the incorrect placement of samples on guide trees. However, except for the excess of over-splits, the effect of unknown guide tree was minimal. The number of exact matches was not significantly different between known and unknown guide tree simulations, and even when DNA sequences were used, accuracy was comparable to the other simulations with a sufficient number of loci. It appears that, when the consensus species tree estimation can resolve a particular node on a guide tree, tr2 does not erroneously merge or split species on the node. This is a useful property because there are discrepancies between the number of loci required for correct delimitation, guide tree estimation, and initial population assignment in the conventional delimitation procedures (Zhang et al. 2014). The inaccurate estimate of guide tree and delimitation may be mediated simply by adding more loci as the number of loci is not a major computational obstacle.

The delimitation results for *Sistrurus* rattlesnakes were partially consistent with the reported results in Kubatko et al. (2011). Though the 2 known taxonomic species, *S. catenatus* and *S. miliarius*, were consistently delimited as putative species, only about 30% of iterations supported the distinctiveness of the subspecies *S.c.catenatus*. Considering the number of loci necessary to delimit species in the simulation studies, 18 nuclear markers appear to be insufficient to fully delimit this group with the present method. The resampling also indicates that polytomies are an important source of uncertainty on delimitations. The 2 alternative outcomes with 18 loci resulted solely from the different resolutions of polytomies. The lack of mutations and resulting polytomies do not positively mislead the delimitation when the identical sequences are randomly inserted or polytomies are randomly resolved. Nevertheless, simulations and case studies show the use of uninformative loci compromise the power of species detection and introduce uncertainty. We used repeated delimitations with randomly resolved gene trees and guide trees, and this approach was able to capture the level of uncertainty of gene tree reconstruction in the rattlesnakes. Resampling trees from bootstrap trees or MCMC runs, as done in the *Bacillus* data set, is an alternative way to handle the uncertainty.

The results of resampling analysis of *Bacillus* complex indicate more uncertainty in their delimitation than the rattlesnakes. The reduced number of species observed on the rooted triple consensus may partly result from the unresolved guide tree due to horizontal transfer between distantly related groups. However, distinctive patterns of bacterial diversification were still observed. Clade A and B were consistently delimited into large groups, whereas clade C mainly consisted of weakly connected singletons. Samples from these 2 categories exhibited a contrasting pattern of linkage disequilibrium patterns. Especially, low LD between loci observed in samples from the largest clusters detected in clades A and B indicates that there is frequent gene exchange between members of those groups. Homologous recombination creates local topological discordance on bacterial genomes (Didelot et al. 2010), and if the recombination events are localized only within closely related groups, the mutually recombining groups can be detected by tr2 through genealogical discordance. The clusters delimited in the clades A and B are likely to be such groups. Clade C has low recombination rates and methods based on recombination and gene congruence are inappropriate. It may still be possible to identify independently evolving groups in such clades using alternative concepts and methods developed for clonal bacteria and asexuals (Cohan 2001; Barraclough et al. 2003). Clearly, the mixture of high- and low-recombining lineages in the *Bacillus* data adds complexities to species delimitation (which we will address in detail elsewhere) and the number of loci may not be large enough to fully elucidate diversification patterns. However, the result demonstrates the potential for detecting “recombinationally isolated” groups in prokaryotes.

The parameters to be considered for the computational complexity of delimitation are the number of samples (K), the number of species (S), and the number of loci (N). The dynamic programming algorithm introduced in this article finds the best delimitation and reduces the complexity of search through a guide tree to time scale linear to S,O(S), which allows a thorough search of a guide tree. For example, using a guide tree that assigns every individual into a distinct species has often been prohibitive with large samples, but, in our simulations, tr2 was able to process guide trees with 100 tips within 150 s. Combined with good performance with respect to other parameters—cubic dependency of time on overall sample size, $O(K^{\text{3}})$ and linear for loci, O(N)—the method could be used to provide a rapid search method through candidate delimitation hypotheses before applying more statistically rigorous methods to large data sets. Current next generation sequencing projects often target a large number of loci from relatively few individuals. The tr2 method is suitable for this type of sampling design because the impact of increasing loci on computations is smaller than increasing individuals. A simulation shows that higher accuracy is achieved with more loci than with more individuals when total sample size (loci × individuals) is fixed (Supplementary Figure S7 in Supplementary Material). This demonstrates a potential use of current sequence technologies for species delimitation though the optimal sampling strategy is yet to be investigated. A final point is that the dynamic programming algorithm introduced in this study may be applied for other optimal partitioning problems using hierarchical structure, such as finding optimal partitioning of sequence alignments for phylogenetic inference (Li et al. 2008; Lanfear et al. 2012).

In this study, we did not consider possible violations of the assumption of the multispecies coalescent model including gene flow between populations. Gene flow between sister species reduces the number of dominant triplets and increases 2 minority triplets equally, and may compromise the accuracy of the method. Incorporating branch lengths into the model may be required to tease apart the effects of gene flow and incomplete linage sorting. Introgression events from distantly related groups may be detected as an increase in 1 of 2 minority triplet counts. Indeed, deviation from equal counts of minority triplets is used for tests of introgressive gene flow (Durand et al. 2011; Zwickl et al. 2014). Violation of the model assumption of panmixia could be detected in a similar manner by extending the trinomial distribution model used in this study to a 3-rate model.

The method now uses estimated gene trees as inputs. In addition, it uses a guide tree estimated from the given gene trees or other independent methods. This procedure does not take the uncertainty of gene tree and species tree inference into account. Also, most computational time of the delimitation procedure was spent on the tree-building steps (a BEAST run on 1 locus of *Bacillus* took 2.5 h, whereas the tr2 ran in 2 min with >100 samples). For guide tree inference, 1 possible solution would be to incorporate joint inference of species tree and delimitation using triplets. Triplet- or quartet-based phylogenetic inference methods using known gene trees under the multispecies coalescent framework have been developed and implementations to handle large data sets already exist (Liu et al. 2010; Mirarab et al. 2014). The delimitation step based on the trinomial distributions could be easily integrated into these procedures. Also, gene tree inference could be bypassed by directly counting triplets estimated from 3 corresponding sequences and an out-group as done in some phylogenetic inference programs (DeGiorgio and Degnan 2010). Combining these methods could potentially lead to a highly scalable joint estimation of species tree and delimitation.

In conclusion, we present a method for species delimitation from multilocus data that can potentially scale to the kind of sample sizes that are currently only feasible for single-locus approaches. The method uses exact methods derived from the multispecies coalescent, but by splitting the problem into triplets it circumvents the computational challenges. As it becomes easier to sequence nonmodel genomes, and consequently to assay variable nuclear markers across clades, we envisage a growth in the number of studies using standardized multiple unlinked markers across entire clades, equivalent to current DNA barcoding sample regimes. Our method is designed with these scenarios in mind to complement more intensive methods.

## Supplementary Material

Supplementary material can be found in the Dryad Data Repository: http://dx.doi.org/10.5061/dryad.3cb25.
